# Olive Leaves Extract and Oleuropein Improve Insulin Sensitivity in 3T3-L1 Cells and in High-Fat Diet-Treated Rats via PI3K/AkT Signaling Pathway

**DOI:** 10.1155/2023/6828230

**Published:** 2023-01-07

**Authors:** Fatma Hadrich, Asma Mahmoudi, Mohamed Chamkha, Hiroko Isoda, Sami Sayadi

**Affiliations:** ^1^Environmental Bioprocesses Laboratory, Sfax Biotechnology Center, P.O. Box 1177, Sfax 3038, Tunisia; ^2^Faculty of Life and Environmental Sciences, University of Tsukuba, Tsukuba, Ibaraki 305-8572, Japan; ^3^Alliance for Research on Mediterranean and North Africa, University of Tsukuba, Japan; ^4^Biotechnology Program Center of Sustainable Development, College of Arts and Sciences, Qatar University, Doha 2713, Qatar

## Abstract

Olive leaves extracts are known to exert potential pharmacological activities especially, antidiabetic and antiobesity. This study explores the anti-insulin resistant effect of olive leaves extracts and oleuropein in 3 T3-L1 cells and in high-fat diet fed rats. Our results showed that ethanol extract (EE) suppressed significantly (*P* < 0.01) triacylglycerol accumulation. In preadipocytes cells, EE 1/100 decreased cell viability and induced apoptosis. Real-time PCR analysis showed that EE reduced the mRNA levels of adipogenesis (CEBP-*α*, PPAR*γ*, SREBP-1c, and FAS) and proinflammatory (TNF-*α* and IL-6) genes. Moreover, the cotreatment of EE 1/1000 or oleuropein with insulin increased considerably the expression of p-IRS, p85-pI3K, and p-AKT. *In vivo* model, the oral administration of oleuropein at 50 mg/kg in rats fed with high fat diet for 8 weeks reduced inflammation in liver and adipose tissues (WAT), improved glucose intolerance, and decreased hyperinsulinemia. Furthermore, the immunohistochemistry revealed that the expression level of p-Akt, IRS1, and Glut-4 were significantly enhanced in liver and WAT tissues after oleuropein supplementation comparing with that in HFD group. Additionally, the expression of IRS1 was markedly ameliorated in pancreas. Our obtained results can be adopted as an approach to used olive leaves as complement to prevent insulin-resistance disease.

## 1. Introduction

Type-2 diabetes mellitus (T2DM), a worldwide chronic metabolic disease, has increased in the last decades. The hallmarks of this global epidemic were a default in insulin secretion and an insulin resistance in the target organs [1]. It has been recognized that there is a close relationship between obesity and T2DM. In this context, many studies have reported that high-fat diet-induced dyslipidemia, chronic inflammation, glucose disorder metabolism, and insulin resistance [2, 3]. In fact, the supplementary fat generated following excess of dietary lipids was translocated into the target tissues including pancreas and liver and consequently caused toxic effects and organs dysfunction. The white adipose tissue (WAT) is an important endocrine organ which secretes a variety of proinflammatory cytokines such as adiponectin, TNF-*α*, MCP-1, IL-2, and IL-6. These hormones regulate the energy metabolism and insulin sensitivity through the modulation of insulin signaling pathway [4, 5]. The phosphatidylinositol 3-kinase (PI3K)/protein kinase B (AKT) signaling pathway has a crucial role in the regulation of glucose metabolism and insulin sensitivity [6]. In fact, the insulin receptor substrate 1 (IRS1) was phosphorylated after binding with insulin and then activates PI3K [7, 8]. Then, the activation of the p110 and p85 subunits induced phosphorylated Akt activation, and allows by transporter glucose-4 (GLUT-4) the glucose translocation. Therefore, the regulation of both TNF-*α* and adiponectin and the regulation of proteins, involved in the insulin pathway, lead to the protection against insulin resistance.

**Figure 1:**
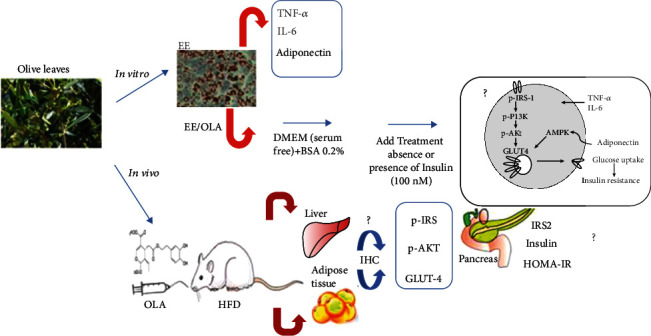
Diagram of the different parameters and mechanisms involved in the experiment.

Numerous antiobesity and antidiabetic drugs have been developed, such as orlistat, sibutramine, and rimonabant. Because of their serious adverse effects, several alternatives are proposed [9]. Recently, natural compounds have been used to treat obesity associated with insulin resistance. In this context, polyphenols and flavonoids, are an abundant plant secondary metabolites, have attracted significant public attention because of their beneficial effects including, scavenging free radicals, deceasing inflammation, and regulating cell signaling pathways [10, 11]. *Olea europaea L.* (Oleaceae) a characteristic Mediterranean specie, is used to produce olives and oil. Olive leaves have been widely used in traditional remedies to treat diabetes, diarrhea, and flatulence. The pharmacological properties of olive leaves are due to their phenolic contents [12]. The antiobesity effect of olive leaves extract (OLE) was earlier demonstrated in high fat diet rats [13]. Similarly, the OLE and its active components such as oleuropein, hydroxytyrosol, apigenin, luteolin, and luteolin-7-O-glucoside, have been explored as a possible diabetes-preventive component in both alloxan and streptozotocin diabetic rats [14–16]. The effect of olive leaves on insulin resistance was not well-established. In our previous study, we investigated the effect of oleuropein on glucose uptake in muscle cells as well as its sensitizing insulin effect [17]. Therefore, we aimed in the current study to investigate the effect of olive leaves extract and oleuropein on insulin sensitivity *in vitro* in 3T3-L1 adipocytes and in high-fat diet-treated rats. Furthermore, we explored the molecular analysis of proteins expression involved in insulin pathway. The diagram in Figure 1 presented the summary of experiments involved in this current study.

## 2. Materials and Methods

### 2.1. Materials

3 T3-L1 cells were provided from the Health Science Research Resources Bank (HSRRB, Osaka, Japan). Fetal bovine serum (FBS), Dulbecco's modified Eagle's medium (DMEM high-glucose), 3-iso-butyl-1-methylxanthine, Dexamethasone, and Insulin were provided from Sigma-Aldrich (Missouri, USA). Adiponectin and TNF-*α* ELISA Kits were purchased from R&D systems. All the primers and antibodies were purchased from Santa Cruz Biotechnology.

### 2.2. Preparation of Olive Leaves Extracts

*Chemlali* olive leaves were collected from Sfax (Tunisia). The leaves were dried into microwave and were powdered. After that, 1 g of olive leaves was extracted with 10 mL ethanol 70% for two weeks in the dark at room temperature to obtain ethanol extract (EE) [18]. The extract was filtered through a 0.22 *μ*m membrane filter (Milllipore, U.S.A) and the supernatant was kept at -80°C until utilization. Oleuropein was purified according to Hadrich et al. [19].

### 2.3. Characterization of Olive Leaves Extract

The identification of phenolic compounds was carried out using a HPLC Agilent 1260 (Agilent Technologies, CA, USA) according to our previous studies [19]. Water with acetic acid (0.1%) (mobile phase A) and acetonitrile (mobile phase B) are the mobile phases. The solvent gradient was monitored according to the following conditions: from 0 to 22 min, 90% A to 50% A; from 22 to 32 min, 50% A to 0% A; from 32 to 40 min, 100% B; and from 40 to 50 min, 0% A to 90% A. The flow rate used was set at 0.50 mL/min.

### 2.4. Effect of Olives Leaves on 3 T3-L1 Cells

10^5^ cell/mL of 3 T3-L1 cells were cultured for two days in DMEM medium with 10% FBS at 37°C and 5% CO_2_. Then, the cells were incubated with a differentiation cocktail (MDI) containing 1 *μ*mol/L dexamethasone and 0.5 mmol/L IBMX and 10 mg/mL insulin for 3 days. 48 h later, the culture medium was changed with medium containing only insulin. The adipocyte maintenance medium was replaced every 2 days until 9 days. To investigate the effect of extracts on cells differentiation, the EE was prepared at final dilution equal to 1/100 and 1/1000 (v/v) in culture media. We used these concentrations according to Ben Othman et al. [18]. A vehicle containing ethanol at 1% was used at the same conditions.

### 2.5. Oil Red O Staining and Lipids Droplets Quantification

At the last day of differentiation, adipocytes cells were stained using adipogenesis kit (Cayman Chemical Company). The cells were checked into microscope to take pictures. The absorbance was measured by a Spectra Max microplate reader (Spectra Max 190; CA, USA) at 540 nm.

### 2.6. Cytotoxic Test

3 T3-L1 fibroblasts cells were seeded in 96-well plate at 10^5^ cell/mL and treated with extracts at 1/1000 and 1/100 dilutions for 24 h. After that, 10 *μ*L of (3-(4,5-Dimethylthiazol-2-yl)-2,5-diphenyltetrazolium bromide (MTT) solution were added. The plate was incubated for 6 h at 5% CO_2_. The medium was removed and 10% sodium dodecyl sulfate (SDS) was added to dissolve the formed formazan. The intensity of the color was quantified at 570 nm. The cell viability percentage was expressed compared to control cells.

### 2.7. ELISA Kits for Adipokines Measurements

Adiponectin and TNF-*α* concentrations in cell culture medium was measured using adiponectin and TNF-*α* ELISA kits according to manufacturers' instructions.

### 2.8. Real-Time Quantitative PCR (qRT-PCR)

The RNeasy Kit (Qiagen) was used to extract RNA from cells. Then, 500 ng of RNA were reverse transcripted. The qRT-PCR was carried out using SYBR green detection of amplified products. Each cDNA was amplified using specific primers at 95°C for 5 s, 60°C for 30 s, and 72°C for 30 s, for 40 cycles). The Table 1 showed the primers used for gene expression. The glyceraldehyde-3-phosphate was used as a reference to normalize the relative expression of target genes.

### 2.9. Effect of Extract and Oleuropein in Insulin Sensitive Cells

After differentiation, adipocytes were washed with DMEM (free serum) and BSA (0.2%) and then, the extract or oleuropein were added. After 24 h, the insulin (100 nm) was added at different times (0, 5, 10, and 30 min).

### 2.10. Western Blotting

Total proteins were extracted by lysis RIPA buffer, and 20 *μ*g were separated with 12% sodium dodecyl sulfate polyacrylamide and then, transferred to polyvinylidene difluoride membrane. After incubation for overnight with primary antibodies (Akt, p-Akt (ser473), IRS, p-IRS1 (ser 307), PI3K, and p85-PI3K) at 4°C, corresponding secondary antibodies were added for 1 h after recovering the specific antibody. The protein band was detected by a chemiluminescence (ECL) detection kit (Amersham Bioscience, NJ). The band intensity was quantified using ImageJ program. *β*-Actin antibody, an internal control, was used.

### 2.11. Effect of Oleuropein in High Fat Diet Induced Insulin Resistance

#### 2.11.1. Animals and Study Design

Male rats weighing about 240 ± 10 g were used. The approval of the experiments was confirmed from the Medical Ethics Committee for the Care and Use of Animals Laboratory of the Pasteur Institute of Tunis (approval number: FST/LNFP/Pro 152012). Briefly, 24 animals were randomly divided in 3 groups: rats fed with standard diet and served as control (CTR), rats fed with HFD (normal diet supplemented with 10% of sheep fat, 5% fructose, and 0.1% bile salts), and rats fed with HFD with daily oral gavage of 50 mg/kg of oleuropein (HFD-OLA) for 8 weeks. After treatment period, animals were sacrificed and blood samples were collected to determine the biochemical parameters and lipid profile. The liver, abdominal fat, and pancreas were excised, weighted, washed, and fixed in 10% neutral buffered formalin (Sigma-Aldrich).

#### 2.11.2. Biochemical Analysis

Triglycerides, total cholesterol, high-density lipoprotein cholesterol, low-density lipoprotein cholesterol, blood glucose, and insulin levels were measured at the clinical and Biochemical Laboratory of the Hedi Chaker University Hospital, Sfax, Tunisia. The estimated insulin resistance was measured using the homeostasis model assessment index for insulin resistance (HOMA-IR)], using the following formula: [fasting glucose (mmol/L) × insulin (mIU/L)/22.5] [13].

#### 2.11.3. Oral Glucose-Tolerance Test and Insulin Tolerance Test

Both oral glucose (OGTT) and insulin tolerance tests (ITT) were carried out after 8 weeks as described by [20]. The rats were fasted for 8-10 h and then, they orally received a solution of glucose (2 g/kg of body weight). The venous blood glucose level was assessed for each group at 0, 15, 30, 60, and 120 min from the tail using a glucometer. Similarly, after overnight fast, a solution of insulin (0.8 U/kg body weight) was injected intraperitoneally for all groups of rats and the blood glucose levels were calculated at 15, 30, 60, and 120 min.

The area under curve (AUC) of glucose was calculated according to the method described by [21].

#### 2.11.4. Hematoxylin Eosin and Immunohistochemistry Analysis

After being paraffin embedding, the sections (4 *μ*m thickness) from each paraffin block were stained with Eosin and Hematoxylin. The size of pancreatic islets was calculated using ImageJ software Immunohistochemistry was performed with specific antibodies against TNF-*α*, p-IRS1, p-Akt, and Glut-4 (Santa Cruz, CA, USA), in order to detect the inflammation and the insulin pathway proteins expression in target tissues (white adipose and liver). The antibodies reactivity was detected using a streptavidin-peroxidase Histostaining-SP kit. Positive immunohistochemistry (IHC) stains were defined as yellow-brown color. The score was determined and expressed according two-variable factors as described by Fki et al. [13].

### 2.12. Statistical Analysis

Statistical data analysis was evaluated using Graph Pad Prism 6.0. The difference between groups was assessed using one-way analysis of variance (ANOVA) followed by Tukey's test for multiple comparison at the level *P* < 0.05. The data are expressed as mean ± standard deviation (SD).

## 3. Results

### 3.1. Characterization of the Extract

The phenolic compounds contained in extracts,were identified based on the retention time and spectral characteristics of their peaks against those of the standards. The major compound in extract present at high amount was oleuropein with a concentration of 15.32 ± 0.3 mg/mL, whereas, the apigenin-7-O-glucoside and luteolin-7-O-glucoside were also found at low quantity, and their concentrations were 1.17 ± 0.06 mg/mL and 1.06 ± 0.05 mg/mL, respectively (data not shown).

### 3.2. Effect of Olive Leaves Rich Extract on 3 T3-L1 Cells

**Figure 2:**
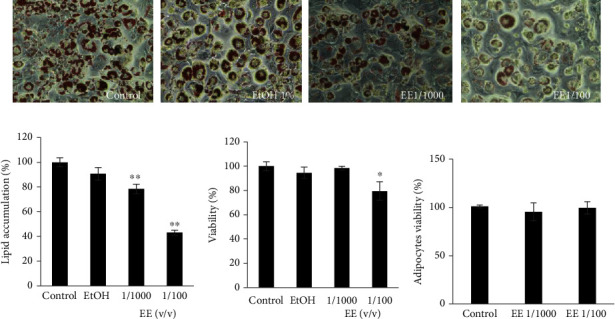
(a) Microscopic observation of lipid droplets after 7 days of differentiation. (b) Quantification of lipid droplets after Oil Red O staining. Lipid droplets in treated cells were expressed as a percentage of control (untreated cells). (c) Effect of olive leaves extracts on 3T3-L1 cell viability after 24 h incubation with cells assessed by MTT assay. (d) Effect of extract on adipocytes viability. Values are expressed as percentage of untreated control cells (mean ± SD). ∗*P* ≤ 0.05 indicate a significant difference compared to control. ∗∗*P* ≤ 0.01 indicates that the mean value is significantly different vs. control.

The lipid accumulation was observed after 7 days of treatment by microscope after Oil Red O staining (Figure 2(a)). The treatment with EE inhibited lipid accumulation in 3 T3-L1 cells in a dose-dependent manner (1/1000 and 1/100). The optical density measured at 420 nm showed a significant reduction (*P* < 0.01) in intracellular triacylglycerol in the presence of olive leaves extract compared to control cells. In fact, lipid accumulation was decreased by 20% ± 4.5 and 57.3% ± 3.7 for EE1/1000 and EE1/100, respectively (Figure 2(b)). The cytotoxic effect of olive leaves extracts was investigated. The cells were treated with various concentrations at 1/1000 and 1/100 for 24 h. As shown in Figure 2(c), EE at 1/100 dilution reduced significantly (*P* < 0.01) cell viability with a percentage up to 20%. Whereas, the ethanol extract at 1/1000 has no toxic effect on cells. On the contrary, both treatments did not influence mature adipocyte viability with EE1/100 (v/v) (Figure 2(d)).

### 3.3. Olive Leaves Extract Regulates Markers of Adipogenesis and Inflammation

**Figure 3:**
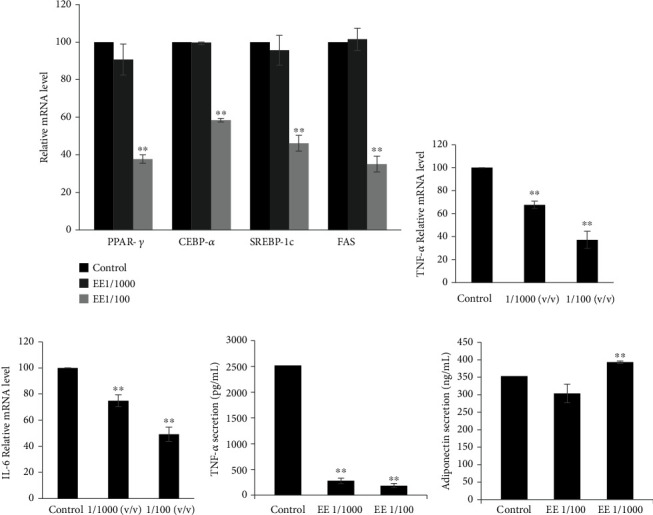
(a) Effect of extracts on gene levels of C/EBP*α*, PPAR*γ*, SREBP-1C, and FAS in 3T3-L1 adipocytes. (b) Effect of olive leaves ethanol extract on mRNA gene levels of TNF-*α*, and IL-6 (c) in 3T3-L1 adipocytes. Effect of extracts on TNF-*α* (d) and adiponectin (e) production in 3T3-L1 adipocytes. The reported values are the means ± SD (*n* = 3). ∗∗*P* ≤ 0.01 indicates a significant difference vs. control.

The adipocyte differentiation-related gene levels were determined using Real-time RT-PCR. The result revealed that the mRNA gene levels of C/EBP*α* and PPAR*γ*, SREBP-1c were remarkably (*P* < 0.01) decreased in the presence of ethanol extract at 1/100 (Figure 3(a)). Our data indicated also that ethanol extract reduced significantly (*P* < 0.01) the mRNA level gene FAS (Figure 3(a)). Additionally, molecular analysis indicated that olive leave extract decreased significantly the mRNA gene levels of TNF-*α* (Figure 3(b)) and IL-6 (Figure 3(c)).

### 3.4. Effect of Extracts on Adipokines Secretion

The effect of extracts on adipokines released was assessed after 9 days of differentiation. The conditioned medium was collected for ELISA assay.  Figure 3(d) showed that both extracts decreased TNF-*α* secretions by 92.67% ± 0.04 and 88.6% ± 0.05, for EE/100 and 1/1000, respectively. However, in the case of adiponectin, treated cells with EE at dilution 1/1000 caused a significant increase (*P* ≤ 0.01) in adiponectin secretion by 1.2-fold compared to the control (Figure 3(e)).

### 3.5. Olive Leaves Extract and Oleuropein Modulated PI3K/Akt Pathway

**Figure 4:**
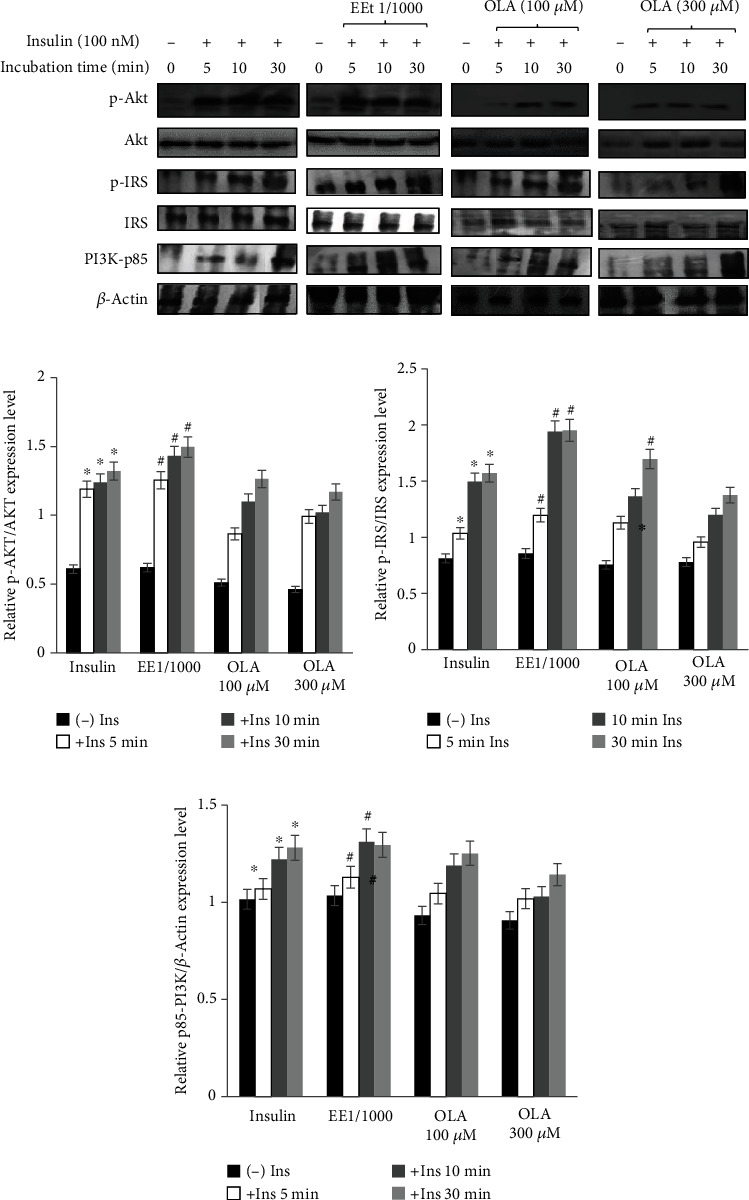
(a) Effect of ethanol extract and oleuropein on the level of p-AKT, p-IRS1, and p85-PI3K protein expression before and after insulin induction. Differentiated 3T3-L1 cells were treated with extract at 1/1000 or oleuropein (100 and 300 *μ*M) for 24 h and then 100 nM of insulin was added for 5, 10 min, and 30. The expression of p-Akt (ser 473) (b), p-IRS (ser 307) (c), p85-PI3K (d), and *β*-actin was quantified. ∗∗*P* < 0.01 indicates a significant difference vs. control (without insulin). ^#^ indicates a significant difference compared vs. insulin alone. ^#^*P* < 0.05.

Our results showed that treatment with ethanol extract or oleuropein (100 *μ*M and 300 *μ*M) in the absence of insulin has no effect on the expression level of p-IRS1, p-Akt, and PI3K-p85 (Figure 4(a)). Surprisingly, the cotreatment with insulin increased significantly the expression of these proteins (*P* ≤ 0.01) and this expression was kept until 30 min. The level of Akt phosphorylation was increased by 1.49, 1.13, and 1.06-fold in ethanol extract at 1/1000, oleuropein (OLA) at 100, and 300 *μ*M, respectively (Figure 4(b)). Similarly, the expression of p-IRS and p85-pI3K were considerably improved after adding oleuropein or olive leaves extract (Figures 4(c) and 4(d)). In fact, after 30 min, the level of IRS expression was increased by 1.95, 1.72, and 1.56 for EE1/1000, OLA100, and 300 *μ*M, respectively. However, the level expression of P85-PI3K was 1.09, 0.99, and 0.88, for EE1/1000, OLA100, and 300 *μ*M, respectively.

### 3.6. Effect of Oleuropein on Body Weight, Glucose Homeostasis, and Insulin Sensitivity

**Figure 5:**
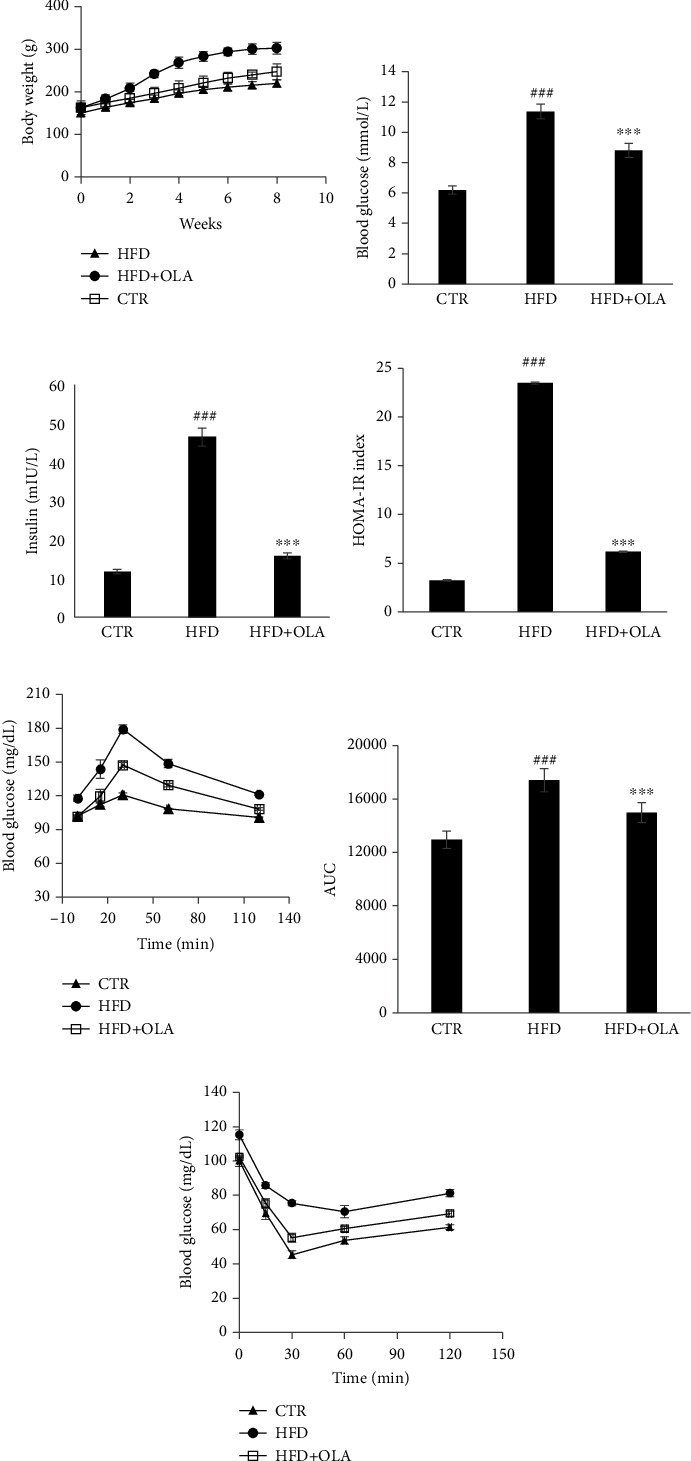
Effect of OLA administration on rat fed with high fat diet. (a) Body weight. (b) Fasting blood glucose levels. (c) Serum insulin content. (d) HOMA-IR. (e) Glucose level in the OGTT. (f) The AUC values of the OGTT. (g) Changes in the glucose level in the ITT. Data are expressed as mean ± SD^#^ indicates a significant difference compared vs. CTR. ^#^*P* < 0.05, ^##^*P* < 0.01, ^###^*P* < 0.001. ∗indicates a significant difference vs. HFD. ∗*P* < 0.05, ∗∗*P* < 0.01, ∗∗∗*P* < 0.001.

After 8 weeks of treatment, the final body weight in the HFD group was significantly increased by 37.55% compared with the control group (Figure 5(a)). However, the administration of oleuropein decreased the body weight by 18.1% compared with that in HFD group. Moreover, serum glucose was higher in HFD treated group. In fact, the concentrations were 11.36 mmol/L and 6.1 mmol/L in HFD and control groups, respectively (Figure 5(b)). However, the supplementation of oleuropein recovered this hyperglycemia and maintained the concentration at 8.8 mmol/L. Similarly, feeding rats with oleuropein at 50 mg/mL reduced considerably insulin content as well as the HOMA-IR (Figures 5(c) and 5(d)). These results can be due to the suggested enhancement of pancreatic function, that OLA could alleviate the insulin resistance and improved insulin sensitivity.

### 3.7. Effect of Oleuropein on OGTT and ITT

After administration of glucose, the blood glucose level increased rapidly up to 30 min in HFD group comparing to control group and returned then to their initial value at 120 min (Figure 5(e)). In fact, the blood glucose concentration at 30 min was 179.25 and 120.75 mg/dL for HFD and control groups, respectively. After oral administration of oleuropein, the glucose level peak was reduced by 17.8% at 30 min. Similarly, the AUC values of the OLA groups were decreased significantly by 14.2% (*P* < 0.05), compared with the corresponding AUC of HFD group (Figure 5(f)).

Additionally, after 6 weeks, the insulin was injected to all groups. Our results revealed that after insulin injection, the blood glucose level measured in all groups decreased rapidly, and reached the lowest level at 30 min (Figure 5(g)). The glucose level was increased to the normal state within 60-120 T after oleuropein administration at 50 mg/kg. Our findings are in lines with OGTT and serum parameters and confirmed that oleuropein improved glucose tolerance and alleviate insulin resistance.

### 3.8. Effect of OLA on Serum Lipid Profiles

Table 2 illustrated the serum concentrations of TG, TC, LDL-C, and HDL-C in different groups of rats. As we can see, HFD group presented high serum levels of TG, TC, and LDL-C compared with untreated group (*P* < 0.05). However, oleuropein treated group (50 mg/kg) showed considerably reduced serum contents of TC, TG, and LDL-C and increased HDL-C contents, compared with HFD group (*P* < 0.05). These data indicated that the administration of OLA could regulate hyperlipidemia in HFD rat.

### 3.9. Effects of Oleuropein on Inflammation and Markers Modulating Insulin Resistance and Glucose Transportation in Pancreas, Liver, and Adipose Tissues

**Figure 6:**
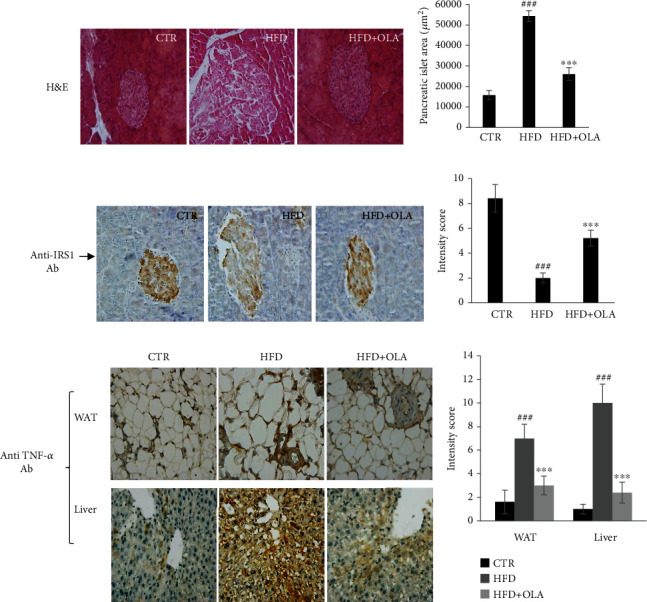
Effect of oleuropein treatment on pancreatic function and inflammation in target tissues and of HFD-fed rats. (a) H&E-stained pancreatic sections. (b) Pancreatic islet size (*μ*m^2^). (c) Representative image of immunostaining for IRS1 in pancreas. (d) Immunostaining intensity score. (e) Immunohistochemical staining with anti-TNF-*α*, in liver, and white adipose tissues of the different groups. (f) Immunostaining intensity score of TNF-*α*. Control group (CTR), treated group with HFD (HFD), treated group with HFD, and oleuropein (HFD + OLA). All values are expressed as the mean ± SD ^#^ indicates a significant difference compared vs. CTR. ^#^*P* < 0.05, ^##^*P* < 0.01, ^###^*P* < 0.001. ∗indicates a significant difference vs. HFD. ∗*P* < 0.05, ∗∗*P* < 0.01, ∗∗∗*P* < 0.001.

The H&E results showed a compensatory increase of the pancreatic islet size and vacuolization in the HFD group compared with that in the control group (CD) (*P* < 0.05) (Figure 6(a)). OLA supplementation considerably reduced the pancreatic islet area (Figure 6(b)). The immunohistochemistry results (IHC) showed that compared with HFD group, the expression of IRS1 was significantly increased after OLA administration (Figures 6(c) and 6(d)). Additionally, the IHC analysis revealed that expression of the TNF-*α* proinflammatory cytokine was markedly reduced in white adipose (WAT) and liver tissues of rats treated with OLA (*P* < 0.05) confirming the anti-inflammatory effect of oleuropein (Figurse 6(e) and 6(f)).

**Figure 7:**
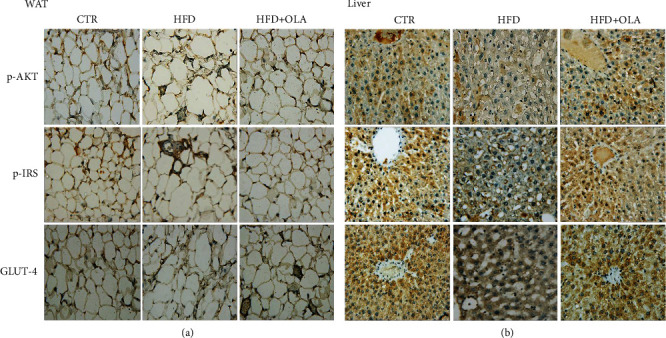
Representative images of immunostaining for p-IRS1, p-AKT, and GLUT4 in the abdominal fat tissue (1000× magnification) (a) and liver tissue (1000× magnification) (b) of CTR (control group), HFD-fed rat and treated HFD with OLA.

The effect of the HFD and the oleuropein on proteins involved in insulin pathway showed that the proteins levels of p-IRS1, p-AKT, and Glut-4 were reduced in the liver and the white adipose tissues of HFD group. However, after oleuropein treatment, the expression of these proteins was increased significantly (*P* < 0.05) (Figures 7(a) and 7(b)). The IHC score of the different antibodies in WAT and liver tissues were presented in Tables 3 and 4, respectively. Our data are in agreement with the results obtained in 3 T3-L1 cells that OLA ameliorate insulin sensitivity by activating the PI3K/AKT signaling pathway.

## 4. Discussion

Many studies have revealed a wide spectrum of biological activities of olive leaves and its compounds against diabetes induced by alloxan, however, few studies have focused on their protective effect on Type-2 mellitus caused by obesity. In this work, we examined the effect of olive extract, prepared as a decoction, and its major compound, the oleuropein on insulin resistance induced in adipocytes cells and in rats fed with high-fat diet. Our results revealed that ethanol extract was effective on triacylglycerol suppression in 3 T3-L1 cells. In fact, it inhibited lipid droplets accumulation by around 40 and 70% at 1/1000 and 1/100, respectively. As we described earlier, EE contained the oleuropein as the major compound with a mixture of flavonoids at lower quantity like apigenin-7-O-glucoside and luteolin-7-O-glucoside. Drira et al. [22] have documented that oleuropein and hydroxyrtyrosol inhibited lipid accumulation and the percentage of inhibition is lower than ethanol extract. Similarly, Shen et al., [23] have demonstrated that olive leaves extract exerted beneficial effects of weight reduction better than those exhibited by oleuropein-supplemented diet. This suggests the synergistic effect between phenolic compounds presents in extracts. Additionally, some olive leaf flavonoids such as apigenin, luteolin, and their derivates have been reported to reduce intracellular lipid accumulation. Previous reports indicated that preadipocytes acquire a relative resistance to apoptosis when they differentiate [24]. Our results of the MTT assay of 3 T3-L1 preadipocytes indicated that EE caused a decrease in preadipocytes viability, up to 15% at 1/100 dilution, which can eventually lead to apoptotic effect. In this context Stefanon and Colitti have demonstrated that hydroxytyrosol induced apoptosis in preadipocytes cells after 10 and 20 days of Omental Differentiation Medium (OM-DM) [25].

During adipogenesis, PPAR*γ*, and C/EBP*α* are the key transcription factors involved in adipocyte differentiation and they play important role in lipogenesis. In fact, many researchers have reported that the modulation of these factors improve the antiobesity effect. Several reports have demonstrated that olive leaves extract inhibits PPAR*γ* and C/EBP*α* in vitro model and in high-fat diet-induced obesity. Indeed, Drira et al., have demonstrated that oleuropein the major compound found in extract exerted antiadipogenic effect by inhibition of these factors, but it remained lower than our results which could be explained by the synergistic effect between extract compounds. In this context, Olive leaves constituents have been reported to improve obesity management by reducing intralipid accumulation in adipocytes cells and in vivo model [26, 27].

During obesity, various adipokines and proinflammatory cytokines are generated through the activation of stress pathway [28]. In fact, many studies have reported that HFD induced the increase of TNF-*α* plasma level. The recruitment of macrophages is the main characteristic of inflammation in white adipose tissue and promoted the secretion of TNF-*α*, IL-2, and IL-6 which cause insulin resistance [4]. Moreover, adiponectin regulates the energy metabolism mainly by increasing insulin sensitivity. Our data indicated that treated 3 T3-L1 adipocytes with ethanol extract downregulated the level genes of TNF-*α* and IL-6. However, it increased efficiently the adiponectin secretion. These data were confirmed *in vivo* model in HFD fed rats where the administration of oleuropein reduced the expression of TNF-*α* in WAT. In fact, our outcomes are in line with the report of Liu et al., who described the efficient inhibitory effect of olive leaves extract and the oleuropein against the expression of proinflammatory cytokines in diabetic rats induced by streptozotocin [29]. Furthermore, in our previous *in vivo* study, we demonstrated that oleuropein increased plasma adiponectin in high cholesterol diet induced obesity in rats [30]. Similarly, Scoditti et al. have reported that hydroxytyrosol alone or in combination with oleic acid significantly prevented TNF-*α*-induced suppression of total adiponectin secretion [31].

As it was reported earlier, the HFD induced dyslipidemia and insulin resistance. In this present study, we demonstrated that the administration of oleuropein decreased serum perturbations induced by HFD. The cholesterol and triglycerides were significantly decreased in OLA treated groups. Particularly, the blood glucose and insulin levels were decreased in rats administrated with OLA. These results confirmed the results of Fki et al. who have revealed that OLA (16 mg/kg) exerted hypoglycemic effect [13]. Similarly, our OGTT and ITT results confirmed that oleuropein alleviated glucose intolerance and insulin resistance. Recent study of Haidari et al. have demonstrated that the supplementation of olive leaves extract capsule comprised 50 mg oleuropein reduced insulin level in adults obese women and improve insulin sensitivity by increasing adiponectin secretion [32]. Additionally, Zhang et al., have revealed that the administration of oleuropein attenuate glucose level and enhanced insulin sensitivity in gestational diabetes mellitus (GDM) mice [33].

Several researchers have reported that the expansion of *β*-cell and pancreatic islet hypertrophy occurred in high fat diet rat with insulin resistance. The increase of insulin secretion associated with the decrease of the IRS1 expression level in HFD treatment proved pancreas dysfunction. In fact, insulin pathway plays a central role in pancreatic *β*-cells. IRS1 and IRS2 are indispensable for insulin biosynthesis and *β*-cell proliferation, respectively [34]. The decreased mean size of islet, the reduced level of insulin, and the enhancement of the IRS-1 expression level after treatment with OLA, suggest an improvement in pancreatic islet function and consequently an enhancement in insulin sensitivity. Our data are consistent with the findings of Lepore et al., who have explored the insulin sensitizing effect of oleacein and oleuropein at 20 mg/kg via ERK pathway activation [35].

To confirm these results, it is necessary to explore the effect of extract and oleuropein on the expression of proteins involved in insulin pathway. The Akt is a central mediator of many metabolic actions of insulin [36]. Our findings proved that in the absence of insulin, the EE or pure oleuropein had no effect on PI3K/Akt pathway protein expression. However, the cotreatment with insulin and olive leaves compounds increased the level of p-IRS, p-Akt, and p85 proteins expression and enhanced the glucose consumption. The level of phosphorylation in the case of extract EE1/1000 was higher than in the presence of oleuropein. Similarly, the expression of p-IRS and p85-PI3K were increased significantly with the cotreatment of insulin and olive leaves. Few studies have focused on the effect of olive leaf extract on insulin sensitivity in adipocytes cells. In C2C12 cells, Fujiwara et al. [37] have reported that treated cells with oleuropein alone at low concentration (10 *μ*M) have no effect on p-Akt. However, the cotreatment with insulin increased the phosphorylation of p-Akt [37]. Other researchers have demonstrated the effect of plant extract on insulin sensitivity in 3T3-L1 adipocytes cells. In this sense, Kalekar et al., showed that curcuma longa extract (Cl) in combination with insulin showed an increase in the glucose uptake after 20 minutes and they suggest that this extract could be beneficial in in treating type II diabetes [38]. Similarly, Zhu et al. [39] have reported that preincubation with apelin increased adipocytes' insulin-stimulated glucose uptake and improved PI3K/Akt pathway after cotreatment with insulin.

These *in vitro* findings were further confirmed *in vivo* model in high fat diet induced insulin resistance. Indeed, the immunohistochemistry showed that rats fed with HFD presented decreased level of p-IRS1, p-AKT, and Glut-4. Additionally, the supplementation of oleuropein increased the expression level of proteins included in signaling pathway and enhanced the glucose translocation into cytoplasm by increasing the expression of Glut 4 compared with HFD group. Our results were an agreement with the report of Fujiwara et al. [37] proved that oleuropein induced GLUT4 translocation in C2C12 muscle cells and gastrocnemius muscles of mice fed with high fat diet. In addition, previous *in vitro* researches have established that the antilipogenesis, anti-insulin resistance, and anti-inflammatory effects of oleuropein observed by microarray analysis are likely to contribute to the improvement of diabetic phenotypes [40].

## 5. Conclusion

In this study, we demonstrated that olive leaves extract decreased the intracellular lipids in 3 T3-L1 adipocytes, and suppressed the mRNA genes level of inflammation (TNF- *α* and IL-6). Moreover, extract 1/1000 increased the release of adiponectin in 3 T3-L1 cells. The cotreatment of extract or oleuropein with insulin increased the expression of phosphorylated IRS1, PI3K, and Akt proteins. The oral administration of oleuropein improved glucose tolerance and reduced insulin content in high fat diet induced insulin resistance in rats. Moreover, the supplementation with OLA reduced inflammation, reduced pancreas islet area and increased the expression of p- IRS1, p85-PI3K, p-Akt, and Glut-4 in target tissues. These findings may provide the consumption of olive leaves extract at low dose of dilution or 50 mg of oleuropein as a food supplement for the prevention of type 2 diabetes.

## Figures and Tables

**Table 1:** Primers for RT-PCR. PCR was performed using the primers indicated as below under optimal amplification condition (95°C for 5 min; 22–35 cycles of 95°C for 30 s, 58°C for 30 s, 72°C for 30 s; and 72°C for 7 min) for each gene.

Name	Forward	Reverse
GAPDH	5′-TGGTGAAGGTCGGTGTGAACGG-3′	5′-TGCCGTTGAATTTGCCGTGAGT-3′
PPAR*γ*	5′-AAACTCTGGGAGATTCTCCT-3′	5′-TGGCATCTCTGTGTCAAC-3′
C/EBP*α*	5′-GCCAAACTGAGACTCTTC-3′	5′-TGGCATCTCTGTGTCAAC-3′
SREBP-1c	5′-GCTTAGCCTCTACACCAACTGGC-3′	5′-ACAGACTGGTACGGGCCACAAG-3′
FAS	5′-TGGAGCCTGTGTAGCCTTCGAG-3′	5′-ACAGCCTGGGGTCATCTTTGCC-3′
Il-6	5′-GGTGACAACCACGGCCTTCCC-3′	5′-GCCACTCCTTCTGTGACTCCAGC-3′
TNF-*α*	5′-AAATGGGCTCCCTCTCATCAGTTC-3′	5′-TCTGCTTGGTGGTTTGCTACGAC-3′

**Table 2:** Effect of oleuropein administration on serum lipids profiles.

Parameters	CTR	HFD	HFD + OLA
TC	0.95 ± 0.07	1.91 ± 0.20^###^	1.48 ± 0.12^∗∗∗^
TG	1.27 ± 0.1	3.68 ± 0.27^###^	1.65 ± 0.125^∗∗∗^
LDL-C	0.38 ± 0.08	0.88 ± 0.045^###^	0.67 ± 0.055^∗∗∗^
HDL-C	0.61 ± 0.06	0.19 ± 0.09^###^	0.43 ± 0.085^∗∗∗^

CTR (control group rats fed a normal diet), HFD: rats fed high-fat diet, HFD + OLA: rats fed high-fat diet with oleuropein (50 mg/kg). Values are expressed as means ± SD. “Significant differences were observed between the CTR and HFD groups: ^#^*p* < 0.05; ^##^*p* < 0.01; ^###^*p* < 0.001. Significant differences were observed between the HFD and the HFD + OLA groups: ^**^p < 0.01; ^***^p < 0.001.

**Table 3:** Scoring criteria of immunohistochemistry assay with antibodies including in signaling pathway in WAT tissue.

p-AKT						
Staining positive cells			Staining intensity		Final score product	
Groups	Percent%	Score 1	Intensity	Score 2	Score 1 X Score 2	Score 3
CTR	51%-75%	3	Strong	3	9-12	3+(+++)
HFD	6%-25%	1	Weak	1	2-4	1+(+)
HFD + OLA	26%-50%	2	Moderate	2	5-8	2+(++)
p-IRS						
CTR	51%-75%	3	Strong	3	9-12	3+(+++)
HFD	6%-25%	1	Weak	1	2-4	1+(+)
HFD + OLA	26%-50%	2	Moderate	2	5-8	2+(++)
GLUT-4						
CTR	51%-75%	3	Strong	3	9-12	3+(+++)
HFD	<5%	0	Absent	0	0-1	0(-)
HFD + OLA	6%-25%	1	Weak	1	2-4	1+(+)

Note: scoring results are determined by screening 12 consecutive microscopic fields. Percent positive cells (score 1) multiply staining intensity (score 2) equals to final product score (score 3). Either 0 or (-) depicts negative staining. An individual slide had <5% of staining cells (= 0) with a staining intensity of 1 (= weak) which would generate a final product score of 0 × 1 = 0; another slide had 80% of staining cells (= 4) with a staining intensity of 3 (= strong) which would give a final product score of 4 × 3 = 12 (3+).

**Table 4:** Scoring criteria of immunohistochemistry assay with antibodies including in signaling pathway in liver tissue.

p-AKT						
Staining positive cells			Staining intensity		Final score product	
Groups	Percent%	Score 1	Intensity	Score 2	Score 1 X Score 2	Score 3
CTR	26%-50%	2	Moderate	2	5-8	2+(++)
HFD	6%-25%	1	Weak	1	2-4	1+(+)
HFD + OLA	26%-50%	2	Moderate	2	5-8	2+(++)
p-IRS						
CTR	51%-75%	3	Strong	3	9-12	3+(+++)
HFD	<5%	0	Absent	0	0-1	0(-)
HFD + OLA	51%-75%	3	Strong	3	9-12	3+(+++)
GLUT-4						
CTR	51%-75%	3	Strong	3	9-12	3+(+++)
HFD	<5%	0	Absent	0	0-1	0(-)
HFD + OLA	51%-75%	3	Strong	3	9-12	3+(+++)

Note: scoring results are based on screening 12 consecutive microscopic fields.

## Data Availability

The datasets used and/or analyzed during the current study are available from the corresponding authors on reasonable request.

## Abbreviations

Akt: Proteine kinase B
C/EBP*α*: CCAAT enhancer binding protein alpha
DMEM: Dulbecco's modified Eagle's medium
DEX: Dexamethasone
EE: Ethanol extract
FAS: Fatty acid synthase
GAPDH: Glycerol-3-phosphate dehydrogenase
GLUT-4: Transporter glucose-4
H&E: Hematoxylin and eosin
HFD: High fat diet
IBMX: 3-Isobutyl-1-methylxanthine
IL-6: Interleukin 6
IHC: Immunohistochemistry
IRS: Insulin receptor substrate
OLA: Oleuropein
PI3K: Phosphatidylinositol 3 kinase
PPAR*γ*: Peroxisome proliferator-activated receptor-gamma
SREBP-1c: Sterol regulatory element-binding proteins
TNF-*α*: Tumor necrosis factor
T2DM: Type 2 diabetes mellitus.
